# New Insights on the Mechanism of Fatty Acids as Buccal Permeation Enhancers

**DOI:** 10.3390/pharmaceutics10040201

**Published:** 2018-10-24

**Authors:** Cristina Padula, Silvia Pescina, Sara Nicoli, Patrizia Santi

**Affiliations:** Department of Food and Drug, University of Parma, Parco Area delle Scienze 27/a, 43124 Parma, Italy; cristina.padula@unipr.it (C.P.); silvia.pescina@unipr.it (S.P.); sara.nicoli@unipr.it (S.N.)

**Keywords:** buccal mucosa, structure-activity relationship, fatty acids, penetration enhancers, dextran, lipophilicity

## Abstract

Buccal mucosa has recently received much attention as a potential route for systemic delivery of drugs, including biologics and vaccines. The aim of this work was to gain insight into the mechanism of fatty acids as buccal permeation enhancers, by studying the effect of a series of medium and long chain fatty acids on the permeation of a model high molecular weight and hydrophilic molecule, fluorescein isothiocyanate labelled dextran (FD-4, m.w. 4 kDa) across porcine esophageal epithelium. A parabolic relationship between fatty acid lipophilicity and enhancement was obtained, regardless of the presence and number of double bonds. The relationship, which resembles the well-known relationship between permeability and lipophilicity of transdermal delivery, presents a maximum value in correspondence of C10 (logP *approx.* 4). This is probably the ideal lipophilicity for the fatty acid to interact with the lipid domains of the mucosa. When the same analysis was performed on skin data, the same trend was observed, although the maximum value was reached for C12 (logP *approx.* 5), in agreement with the higher lipophilicity of the skin. The results obtained in the present work represent a significant advancement in the understanding of the mechanisms of action of fatty acids as buccal penetration enhancers.

## 1. Introduction

Buccal mucosa has recently received much attention as a potential route for systemic delivery of drugs, including biologics [1] and vaccines [2]. Compared to the other routes of administration, the buccal has some advantages in terms of accessibility, high vascularization with direct drainage in the jugular vein which means the avoidance of first pass effect, and low enzymatic activity [3]. On the other hand, the small area available for absorption and the low permeability of the mucosa require the use of appropriate absorption enhancement strategies to obtain suitable permeation profiles, particularly in the case of high molecular weight drugs [4,5]. To date, the most investigated strategy of enhancing buccal absorption is based on the use of chemical permeation enhancers [6,7]; surfactants, cyclodextrins, terpenes, bile salts, chitosan and, more recently, amino acids have been successfully used to promote the buccal permeation of a number of molecules. One of the concerns in the use of chemical permeation enhancers, in particular surfactants and bile salts, is their potential toxicity. Fatty acids, endogenous molecules extensively investigated for their efficacy in promoting transdermal delivery, act primarily through an interaction with the lipid components of the stratum corneum [8]. Despite the interest obtained in transdermal delivery, they have received a limited attention for buccal delivery, concentrated mainly on oleic acid; oleic acid was successfully used to increase the permeation of small molecules such as propranolol [9], buspirone [10] and lidocaine [11], whereas it was ineffective in the case of 5-FU [12] and didanosine [13]. Regarding molecules with higher molecular weight, the literature is even more limited [4]. Oleic acid, in combination with PEG200, was able to significantly increase the permeation of a model peptide of about 570 Da across porcine buccal mucosa from a cubic phase of glyceryl monooleate and water [14]. The presence of oleic acid in a poloxamer gel produced an increase of the hypoglycemic effect of insulin administered via buccal route in rats [15]. Finally, the pre-treatment of the hamster buccal mucosa with cod-liver oil extract, containing 16 different fatty acids, produced an increase in the permeation of ergotamine [16]. It should be noted that the latter two examples used animal models with keratinized buccal mucosa, in contrast to the buccal mucosa of humans [6]. Concerning the mechanism of action, the following have been proposed, although without direct evidence [6]: change of membrane fluidity, membrane destabilization due to cholesterol dissolution, disturbance of the lipid packing [4] and increase in partitioning. The latter can be of considerable influence in the case of basic permeants, such as propranolol [17], metaproterenol [18] or naphazoline [19], which can interact with fatty acids forming ion pairs, whose partitioning in the lipophilic barrier is increased.

The aim of this work was to gain insight into the mechanism of fatty acids as buccal permeation enhancers, by studying the effect of a series of medium and long chain fatty acids on the permeation of a high molecular weight and hydrophilic model molecule, fluorescein isothiocyanate labelled dextran (FD-4, m.w. 4 kDa), across porcine esophageal mucosa, an accepted model of human buccal mucosa [20].

Taking as a reference the factors governing the activity of fatty acids on the skin, the effect of concentration, chain length and number of double bonds was examined. Saturated fatty acids from C6 to C18, and C18 unsaturated fatty acids, with 1, 2 or 3 double bonds, were applied to the epithelium as pretreatment in ethanol solution, at concentration 0.5–15%, corresponding to the application of 0.33–5.0 mg/cm^2^ of fatty acid. This application procedure was chosen because it allows for a direct assessment of the enhancer activity, avoids drug-enhancer interactions and reduces the risk of mucosa damage.

## 2. Materials and Methods

### 2.1. Materials

Fluorescein isothiocyanate labeled dextran of 4 kDa molecular weight (FD-4), caproic acid (C6), caprylic acid (C8), capric acid (C10), linoleic acid (C18:2) and linolenic acid (C18:3) were purchased from Sigma-Aldrich (St. Louis, MO, USA). Lauric acid (C12) was from Merck (Darmastdt, Germany), stearic acid (C18) from ACEF (Fiorenzuola d’Arda, Italy) and oleic acid (C18:1) from Alfa Aesar (Karlsruhe, Germany).

All reagents and chemicals were used as received and were of analytical grade.

### 2.2. Enhancers Tested

The characteristics of the enhancers tested in this work are reported in Table 1. Eight fatty acids were selected in this work: 4 medium chain (C6, C8, C10 and C12) and 4 long chain fatty acids, of which 1 was saturated (C18:0) and 3 were unsaturated (C18:1, C18:2 and C18:3).

### 2.3. Permeation Studies

In vitro permeation studies were conducted across porcine esophageal epithelium. Pig esophagi (Large White or Landrace pigs, age: 11–12 months, weight: 145–190 kg) were obtained from a local slaughterhouse within 2 h from the animal sacrifice. The esophageal mucosa was separated from the outer muscle layer with a scalpel and the epithelium was peeled off from the connective tissue after immersion in distilled water at 60 °C for 60 s [22]. Samples obtained were frozen until use, which occurred within 3 months [23]. The tissue was mounted, using a regenerated cellulose filter (0.45 μm, pore size) as inert support, in Franz’s type diffusion cells (DISA, Milan, Italy), with a diffusion area of 0.6 cm^2^ and a receptor volume of about 4 mL filled with pH 7.4 PBS.

20 µL of fatty acid ethanol solution (fatty acid concentration ranging from 0.5 to 15% *w*/*v*), were applied, in non-occluded conditions, to the epithelium in correspondence of the available diffusion area. After 1 h pretreatment, the donor compartment was filled with 400 µL of FD-4 solution in PBS pH 7.4 (2 mg/mL) and the diffusion of the permeant was monitored up to 5 h. As control, a pretreatment with ethanol 95% was used. A passive diffusion experiment was also performed, without any pretreatment.

### 2.4. Assay of FD-4

The concentration of FD-4 in samples was determined using a Spark multimode microplate reader (TECAN, Mannendorf, Switzerland). The excitation and emission λ were 490 and 535 nm, respectively. The method was specific and the detector response was linear up to 5 µg/mL with a LOQ of 0.01 µg/mL. Blank experiments ensured no interference of the formulation on FD-4 analysis in the receptor solution.

### 2.5. Data Analysis

The cumulative amount of FD-4 recovered in the receptor phase was plotted versus time. The flux of FD-4 across the mucosa (J, µg/cm^2^·h) was calculated as the slope of the regression line at steady state, while the apparent permeability coefficient (P, cm/h) was calculated at steady state as:

P = J/C_D_ (1)

where C_D_ is the concentration of FD-4 in the donor formulation (2 mg/mL).

The enhancement factor (EF) was calculated as the ratio of the permeability coefficients of the permeant in the presence and absence of the fatty acids (passive).

The significance of the differences among the results was assessed using one-way ANOVA followed by a Bonferroni test. All data are reported as mean ± SEM (*n* = 3–6).

## 3. Results and Discussion

*In vitro* evaluation of the effect of fatty acids on FD-4 permeation was performed on porcine esophageal epithelium, from a 2 mg/mL solution in pH 7.4 PBS [24]. Porcine buccal mucosa, in reason of its availability, is an accepted model for human buccal mucosa; however, it is frequently damaged by mastication and its separation from the underlying muscular tissue is not easy. For these reasons, the esophageal porcine mucosa was proposed and characterized [25] as an alternative to buccal porcine mucosa: it is easier to prepare and less damaged by chewing. Lipid characterization and permeation studies with different molecules showed that it is a suitable model for buccal human mucosa [23,26,27]. The permeability coefficient of FD-4 across porcine esophageal epithelium (the permeation profiles are reported in Supplementary material as Figure S1), obtained in passive conditions, was 0.49 ± 0.15 × 10^−4^ cm/h (equivalent to 1.37 ± 0.43 × 10^−8^ cm/s), in agreement with that obtained across porcine buccal epithelium (1.12 ± 0.69 × 10^−8^ cm/s [24]), which was demonstrated to be comparable to human buccal mucosa [24].The usefulness of porcine esophageal epithelium as an *in vitro* model membrane for buccal drug delivery is therefore confirmed also for FD-4.

Most fatty acids are classified as safe by the FDA and are approved as inactive ingredients in a number of formulations. Table 1 summarizes the characteristics of the fatty acids tested, applied as pre-treatment in ethanol for 1 h.

As pointed out for the skin [28], pre-treatment allows for a direct assessment of the enhancer activity and avoids drug-enhancer interactions, although it might not be easy to use in clinical application. Additionally, ethanol evaporates quickly, leading to the possibility to distinguish between the effect of the enhancer and that of the solvent. The choice of the duration of 1 h is derived from the literature data on the skin [28], indicating that no further enhancement is obtained with longer application and was confirmed in preliminary experiments with 5% lauric acid (reported in Supplementary material as Figure S2). Because ethanol (in co-administration [29,30] and as pre-treatment [31]) can increase the permeability of the buccal mucosa, preliminarily the effect of ethanol pre-treatment was verified. The results obtained, reported in Supplementary material as Figure S1, demonstrate that 1 h of pre-treatment with 20 µL of ethanol in non-occlusive conditions does not modify the permeability of the epithelium.

**Figure 1:**
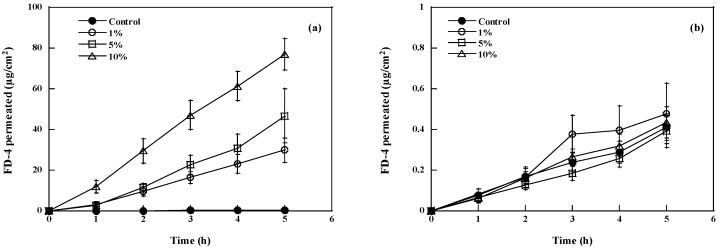
Effect of fatty acid concentration (capric acid, panel (**a**); stearic acid, panel (**b**)) on the permeation of FD-4 across pig esophageal epithelium, compared to the control (ethanol pretreatment). Mean values ± SEM.

Figure 1 shows two examples of FD-4 permeation profiles obtained after pretreatment with increasing concentrations of capric acid (C10, panel a, best case) or stearic acid (C18, panel b, worst case) across pig esophageal epithelium. The permeation profiles were fitted to Equation 1 and the relevant permeation parameters are reported, together with EF, in Table 2. With the exception of caproic and stearic acid, all fatty acids tested were able to significantly increase FD-4 permeation compared to passive, even if to a different extent, in the concentration range explored. The best absolute result was obtained with capric acid (C10) applied at 10% (EF = 148). Among unsaturated fatty acids, the best performing was linolenic acid (C18:3) at 15% (EF = 26).

The comparison of our results with those obtained using bile salts in co-application reveals that bile salts are an enhancer much more efficient for FD-4 delivery than fatty acids, producing EF in the order of 2000 at concentration of 100 mM [32]. However, it should be noted that co-administration leads to the exposure of the mucosa to higher amounts of the enhancer (in the specific case 7 mL of 100 mM solution were in contact with 0.7 cm^2^ of mucosa), compared to pre-treatment (for which a finite amount of enhancer is applied, as indicated in Table 2).

### 3.1. Effect of Fatty Acid Concentration

**Figure 2:**
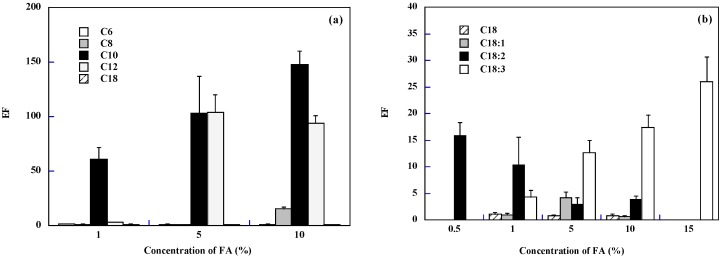
Effect of fatty acid concentration (Panel (**a**) saturated; Panel (**b**) C18 saturated and unsaturated) on the permeation of FD-4 across pig esophageal epithelium. Mean values ± SEM.

Figure 2 reports the EF of FD-4 (calculated as the ratio between permeability coefficients) for the fatty acids studied, grouped in saturated (panel a) and unsaturated (panel b). The effect of fatty acid concentration on FD-4 enhancement across buccal epithelium was not the same for the different fatty acids. For some of them (C6 and C18) there was no effect at any concentrations, for others (C8, C10, C18:3) the EF increased with concentration, for linoleic acid (C18:2) the efficacy decreased with concentration and with lauric (C12) and oleic (C18:1) acids there seems to be an optimal concentration for maximum enhancement.

Considering saturated fatty acids (panel a), the EF increased with concentration although it leveled off (EF *approx.* 100) for lauric acid (C12). This has been observed also in the skin [33], in experiments in which lauric acid was co-applied with the drug naloxone in the presence of propylene glycol; the enhancement presented a maximum at 20%. When considering unsaturated fatty acids (panel b), the presence of one double bond (C18:1) produced a maximum enhancement (EF = 4) for a concentration of 5%; with two double bonds (C18:2) the enhancement decreased with concentration (max EF was 16 at 0.5%), whereas with three double bonds (C18:3) the enhancement increased with concentration, reaching a max value of 26 at 15%.

In general, the ideal concentration depends strongly on the fatty acid; the best result with saturated fatty acids was obtained with capric acid at 10%, whereas, among unsaturated fatty acids, the best performing was linolenic acid at 15%.

### 3.2. Effect of Chain Length

**Figure 3:**
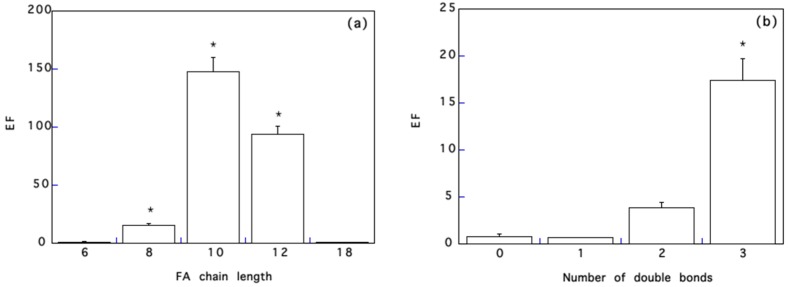
Panel (**a**) effect of fatty acid chain length (applied as pre-treatment at 10%) on FD-4 flux across pig esophageal epithelium. * significantly different among them and compared to the others. Panel (**b**) effect of the number of double bonds of C18 fatty acids (applied as pre-treatment at 10%) on FD-4 permeation across pig esophageal epithelium. * significantly different from the others. Mean values ± SEM.

Figure 3a reports the enhancement factor of FD-4 across porcine esophageal epithelium as a function of the carbon chain length of saturated fatty acids, at a fixed concentration of 10%.

EF augmented as the number of carbon chain increases from 6 to 10 and then decreases, for all concentration tested (see Figure 3a and Table 2): in particular, at 1%, only C10 is active, whereas at 10% the EF follows a parabolic trend with fatty acid chain length, with a maximum always with C10. A parabolic relationship between chain length and enhancement, with an “ideal” chain length corresponding to a maximum of activity, has been identified also with the skin. Aungst et al. [33], for instance, studied the effect of fatty acids in propylene glycol on naloxone permeation across human skin: the maximum enhancement was found for C12. Other authors found maximum efficacies as follows: C9–C10 [34] and C14 [21] for human skin, C12 [35] and C16 [36] for pig skin, C11 for rat skin [35], and C18 for hairless mouse skin [37]. It is likely that C12–C18 chain length (the maximum obtained by most of the authors) corresponds to the optimal balance between permeability of pure acids and affinity for the skin lipids [17,34], whereas in the case of the buccal mucosa, with a higher content of polar lipids [25], a shorter chain length (C10) is required for optimum activity.

### 3.3. Effect of the Number of Double Bonds

Using C18 fatty acids, the effect of the number of double bonds was examined. The results obtained, reported in Figure 3b, indicate that, in analogy with literature data on the skin [33,36,38], the enhancing effect increased with the number of double bonds present. This has been explained considering that the presence of *cis* double bonds causes a kink in the alkyl chain, which can disrupt the stratum corneum lipid packing.

However, examining the combined effect of concentration and number of double bonds one can appreciate other aspects. Oleic acid presents a concentration of max activity (5%), whereas the activity of linoleic acid seems to decrease with concentration and linolenic acid activity increases with concentration (Figure 2b). The literature does not report a systematic analysis of the effect of concentration and number of double bonds, but there are several reports on oleic acid, for which some authors reported a similar behavior for mucosa delivery [9], and for skin delivery [38].

The maximum enhancement obtained with unsaturated fatty acids is smaller compared to the maximum EF obtained with saturated fatty acids of shorter chain length (see Figure 3a). This difference with skin permeation data, where unsaturated fatty acids are generally as active if not more active, can be explained considering that in the mucosa intercellular lipids are loosely packed and less organized than in the skin [39]. Additionally, FD-4 is highly hydrophilic and it is generally assumed that long chain fatty acids are suited to the enhancement of lipophilic drugs, whereas medium chain length fatty acids can be used for both hydrophilic and lipophilic drugs [16,39]. Finally, because the ideal concentration depends strongly on the fatty acid, the optimum concentration (or amount applied) of un-saturated fatty acids might be higher than that applied.

### 3.4. Comparison with Skin Permeation Data and Structure Activity-Relationship

The lipid composition of non-keratinized epithelial barriers, such as the buccal mucosa, is quite different compared to the stratum corneum [40]. In particular, in contrast to the stratum corneum, phospholipids are the most abundant lipids in non-keratinized epithelia. Additionally, epithelia contain mainly glycosylceramides, with only small amounts of ceramides, contrary to the skin.

The comparison of our results with skin permeation data reveals that the experimental conditions are very different. In fact most of the skin permeation data refer to co-administration experiments, in which fatty acids were co-applied with the drug in solutions containing non-volatile co-solvents, which can influence the effect of fatty acids. Additionally, when basic drugs are used, the formation of ion pairs with fatty acids, can induce a further enhancement, due to more favorable partitioning. Concerning the pre-treatment with fatty acids, they are normally applied in occlusive conditions for 1 h [41], 12 h [38] or even 24 h [36].

Despite the differences in permeation barriers, experimental conditions and permeant properties (molecular weight and lipophilicity), our results are in general agreement with skin permeation data, in particular with those of co-administration of naloxone with fatty acids of different chain length and unsaturation in propylene glycol [33], suggesting a common mechanism. Although the main penetration pathway might not be the same (naloxone is much more lipophilic (logP 1.53 [42]) and has a lower molecular weight (327 Da) than FD-4), the effect of fatty acids seems to be mediated by their interaction with barrier lipids, either extraction (in the case of buccal mucosa [6]) or disruption (in the case of skin [42]) of intercellular lipids.

**Figure 4:**
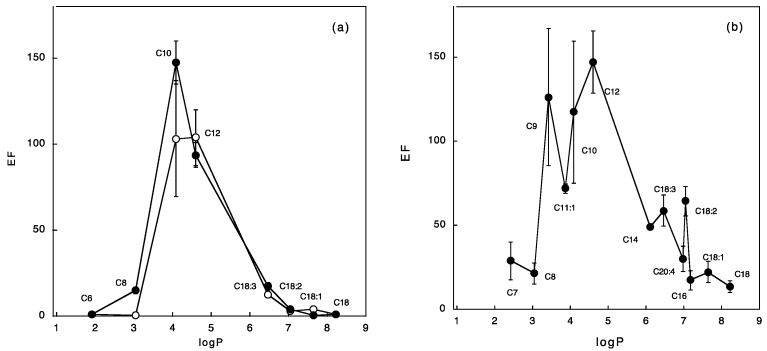
Relationship between fatty acid logP and enhancement factor obtained. Panel (**a**) refers to the present work: empty symbols refer to 5% and full symbols to 10% fatty acid concentration in the pre-treatment solution. Panel (**b**) reports data on naloxone permeation across human skin in the presence of 10% of fatty acids dissolved in propylene glycol [33].

From the above considerations, in an attempt to find a general relationship between the physico-chemical properties of fatty acids and their efficacy in enhancing FD-4 penetration across pig esophageal epithelium, the EF observed was plotted *vs.* the lipophilicity of the acid (calculated logP [21]). The results are reported in Figure 4a, where a parabolic relationship can be observed at 5 and 10% of the enhancer. This result closely resembles the well-known relationship between permeability and lipophilicity of transdermal delivery; the EF increases with fatty acid lipophilicity up to a max value of *approx.* 4, corresponding to C10, and then decreases. This logP value corresponds to the optimal lipophilicity of the enhancer, enabling it to penetrate the mucosa and to interact with its lipid domains.

When the same analysis was performed on the previously cited skin permeation data of naloxone [33] (Figure 4b) a similar trend was observed: again, EF increases with fatty acid lipophilicity to a maximum of *approx.* 5, corresponding to lauric acid (C12). Interestingly, when the same analysis was performed on the data of Reference [35], using a series of fatty acids on melatonin transport across pig skin, the same trend was observed (although the absolute values of enhancement were much smaller), and the EF peaked in correspondence of lauric acid (C12).

In this analysis unsaturated fatty acids fit the general trend, with skin and mucosa, suggesting that partitioning is, if not the most, one of the more relevant properties governing the enhancement. The presence of a different maximum in the two membranes, mucosa and skin, is consistent with the different lipid composition of the two barriers: the stratum corneum is more lipophilic, due to the higher ceramide content, and shows an optimal logP of *approx.* 5, whereas the mucosa is less lipophilic and has 4 as maximum.

## 4. Conclusions

This work reports the first attempt to find a structure-activity relationship on the effect of fatty acids on the mucosa permeation of a high molecular weight hydrophilic model compound (FD-4). A parabolic relationship between fatty acid lipophilicity and enhancement obtained, regardless of the presence and number of double bonds, was found. The relationship, which resembles the well-known relationship between permeability and lipophilicity of transdermal delivery, presents a maximum value in correspondence of C10 (logP *approx.* 4). This is probably the ideal lipophilicity for the fatty acid to penetrate and interact with the lipid domains of the mucosa. When the same analysis was performed on skin data taken from the literature, the same trend was observed, although the maximum value was reached for C12 (logP *approx.* 5), in agreement with the higher lipophilicity of the skin. Finally, the nature of the solvent, together with the type and length of fatty acid chain, may play an important role in the interaction between fatty acids and the intercellular lipids of the tissue, so the effect of co-solvent has to be studied. This work represents a significant advancement in the understanding of the mechanisms of action of fatty acids as buccal penetration enhancers.

## Figures and Tables

**Table 1:** Characteristics of the selected fatty acids.

C:D *	Common Name	IUPAC Name	Formula	m.w.	LogP [21]
6:0	Caproic acid	Hexanoic acid	C_6_H_12_O_2_	116.16	1.92
8:0	Caprylic acid	Octanoic acid	C_8_H_16_O_2_	144.21	3.05
10:0	Capric acid	Decanoic acid	C_10_H_20_O_2_	172.27	4.09
12:0	Lauric acid	Dodecanoic acid	C_12_H_24_O_2_	200.32	4.60
18:0	Stearic acid	Octadecanoic acid	C_18_H_36_O_2_	284.48	8.23
18:1	Oleic acid	(9*Z*)-Octadec-9-enoic acid	C_18_H_34_O_2_	282.46	7.64
18:2	Linoleic acid	(9*Z*,12*Z*)-9,12-Octadecadienoic acid	C_18_H_32_O_2_	280.45	7.05
18:3	Linolenic acid	(9*Z*,12*Z*,15*Z*)-9,12,15-Octadecatrienoic acid	C_18_H_30_O_2_	278.43	6.46

* Number of atoms of carbon (C) and number of double bonds (D) present in the molecule.

**Table 2:** Effect of type and concentration of fatty acid on the permeation parameters of FD-4 across pig esophageal epithelium (mean values ± SEM).

Enhancer Type and Concentration				FD-4 Permeation Parameters			Significativity of Differences
	%	mM	mg/cm^2^	J (µg/cm^2^h)	P × 10^4^ (cm/h)	EF	
Passive	-	*-*	*-*	0.10 ± 0.01	0.49 ± 0.15	-	-
Control	-	-	-	0.11 ± 0.01	0.40 ± 0.04	0.8	-
Caproic (C6)	1	86	0.33	0.16 ± 0.06	0.79 ± 0.28	1.4	not significant
	5	430	1.67	0.11 ± 0.02	0.56 ± 0.11	1.0	
	10	860	3.33	0.13 ± 0.04	0.64 ± 0.18	1.2	
Caprylic (C8)	1	69	0.33	0.13 ± 0.04	0.63 ± 0.22	1.1	*p* < 0.001 vs. 10% *p* < 0.001 vs. 10%
	5	345	1.67	0.05 ± 0.02	0.26 ± 0.09	0.5	
	10	690	3.33	1.67 ± 0.20	8.36 ± 1.02 ^(d)^	15.1	
Capric (C10)	1	58	0.33	6.73 ± 1.21	33.66 ± 6.03	61.0	*p* < 0.05 vs. 10%
	5	290	1.67	11.39 ± 3.72	56.96 ± 18.59 ^(b)^	103.2	
	10	580	3.33	16.31 ± 1.38	81.54 ± 6.92 ^(d)^	147.7	
Lauric (C12)	1	50	0.33	0.30 ± 0.06	1.51 ± 0.32	2.7	*p* < 0.0001 vs. 5 and 10%
	5	250	1.67	11.45 ± 1.81	57.21 ± 9.05 ^(d)^	103.8	
	10	500	3.33	10.35 ± 0.82	51.73 ± 4.09 ^(d)^	93.7	
Stearic (C18:0)	1	35	0.33	0.04 ± 0.04	0.58 ± 0.19	1.0	not significant
	5	175	1.67	0.09 ± 0.02	0.43 ± 0.10	0.8	
	10	350	3.33	0.09 ± 0.03	0.45 ± 0.13	0.8	
Oleic (C18:1)	1	35	0.33	0.09 ± 0.04	0.47 ± 0.19	0.9	*p* < 0.01 vs. 5% *p* < 0.01 vs. 10%
	5	175	1.67	0.45 ± 0.13	2.77 ± 0.53 ^(c)^	4.1	
	10	350	3.33	0.07 ± 0.005	0.37 ± 0.02	0.7	
Linoleic (C18:2)	0.5	18	0.17	1.76 ± 0.64	8.79 ± 1.31 ^(c)^	15.9	*p* < 0.01 vs. 5 and 10%
	1	36	0.33	1.14 ± 0.57	5.71 ± 2.85 ^(a)^	10.3	
	5	178	1.67	0.33 ± 0.13	1.64 ± 0.67	3.0	
	10	356	3.33	0.43 ± 0.06	2.14 ± 0.32	3.9	
Linolenic (C18:3)	1	36	0.33	0.47 ± 0.14	2.35 ± 0.68	4.3	*p* < 0.001 vs. 10 and 15% *p* < 0.05 vs. 15%
	5	180	1.67	1.40 ± 0.26	6.99 ± 1.28 ^(a)^	12.7	
	10	360	3.33	1.92 ± 0.26	9.60 ± 1.29 ^(d)^	17.4	
	15	540	5.00	2.87 ± 0.51	14.37 ± 2.53 ^(d)^	26.0	

Difference with respect to passive: ^(a)^ *p* < 0.05; ^(b)^ *p* < 0.01; ^(c)^ *p* < 0.001; ^(d)^ *p* < 0.0001.

## Supplementary Materials

The following are available online at http://www.mdpi.com/1999-4923/10/4/201/s1, Figure S1: FD-4 permeation profiles across porcine esophageal epithelium without pre-treatment (pas sive) and with ethanol pre-treatment (control) (mean values ± SEM); Figure S2: FD-4 permeation profiles across porcine esophageal epithelium after 1 or 2 h of pre-treatment with lauric acid 5% (mean values ± SEM).
