# Supplementary material for: New Insights on the Mechanism of Fatty Acids as Buccal Permeation Enhancers
Source: Pharmaceutics. 2018 Oct 24;10(4):201. doi: 10.3390/pharmaceutics10040201 (PMC6321376; doi:10.3390/pharmaceutics10040201)
Supplement: Supplementary file 1 [file pharmaceutics-10-00201-s001.pdf]

pharmaceutics

# Supplementary Material: New Insights on the Mechanism of Fatty Acids as Buccal Permeation Enhancers

**Cristina Padula, Silvia Pescina, Sara Nicoli and Patrizia Santi**

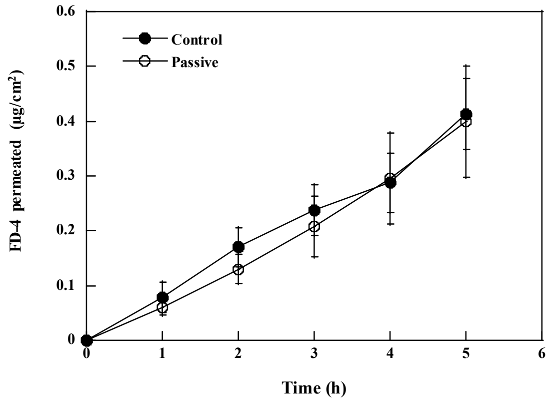


**Figure S1.** FD-4 permeation profiles across porcine esophageal epithelium without pre-treatment (passive) and with ethanol pre-treatment (control) (mean values ± SEM).

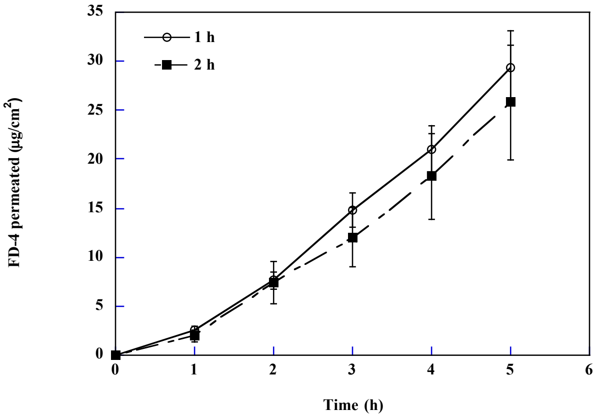


**Figure S2.** FD-4 permeation profiles across porcine esophageal epithelium after 1 or 2 h of pre-treatment with lauric acid 5% (mean values ± SEM).
